# Case report of congenital methemoglobinemia: an uncommon cause of neonatal cyanosis

**DOI:** 10.1186/s40748-022-00142-0

**Published:** 2022-09-16

**Authors:** Allison N. J. Lyle, Rebecca Spurr, Danielle Kirkey, Catherine M. Albert, Zeenia Billimoria, Jose Perez, Mihai Puia-Dumitrescu

**Affiliations:** 1 Division of Neonatal-Perinatal Medicine, Department of Pediatrics, Seattle Children’s Hospital, University of Washington School of Medicine, 4800 Sand Point Way NE, Seattle, WA 98105 USA; 2 Department of Pediatrics, University of Washington School of Medicine, 4800 Sand Point Way NE, Seattle, WA 98105 USA; 3 Division of Pediatric Hematology/Oncology, Department of Pediatrics, Seattle Children’s Hospital, University of Washington, 4800 Sand Point Way NE, Seattle, WA 98105 USA

**Keywords:** Congenital methemoglobinemia, Neonatal, Case report, Hemoglobin M Fort Ripley

## Abstract

**Background:**

Methemoglobinemia can be an acquired or congenital condition. The acquired form occurs from exposure to oxidative agents. Congenital methemoglobinemia is a rare and potentially life-threatening cause of cyanosis in newborns that can be caused by either cytochrome B_5_ reductase or hemoglobin variants known as Hemoglobin M.

**Case presentation:**

A term male infant developed cyanosis and hypoxia shortly after birth after an uncomplicated pregnancy, with oxygen saturations persistently 70–80% despite 1.0 FiO2 and respiratory support of CPAP+ 6 cm H2O. Pre- and post-ductal saturations were equal and remained below 85%. Initial radiographic and echography imaging was normal. Capillary blood gas values were reassuring with normal pH and an elevated pO2. Investigations to rule out hemolysis and end-organ dysfunction were within acceptable range. Given the absence of clear cardiac or pulmonary etiology of persistent cyanosis, hematologic causes such as methemoglobinemia were explored. No family history was available at the time of transfer to our institution. Unconjugated hyperbilirubinemia > 5 mg/dL (442 μmol/L) interfered with laboratory equipment measurement, making accurate methemoglobin levels unattainable despite multiple attempts. Initial treatment with methylene blue or ascorbic acid was considered. However, upon arrival of the presumed biological father, a thorough history revealed an extensive paternal family history of neonatal cyanosis due to a rare mutation resulting in a hemoglobin M variant. Given this new information, hematology recommended supportive care as well as further testing to confirm the diagnosis of congenital methemoglobinopathy. Whole genome sequencing revealed a likely pathogenic variation in hemoglobin. The neonate was discharged home at 2 weeks of age on full oral feeds with 0.25 L/min nasal cannula as respiratory support, with close outpatient follow-up. By 5 weeks of age, he was weaned off respiratory support.

**Conclusion:**

Congenital methemoglobinemia should be considered in the differential diagnosis for newborns with persistent hypoxemia despite normal imaging and laboratory values. Accurate quantification of methemoglobin concentrations is challenging in neonates due to the presence of other substances that absorb light at similar wavelengths, including HbF, bilirubin, and lipids.

## Background

Congenital methemoglobinemia is a rare and potentially life-threatening cause of cyanosis in newborns [1, 2]. Methemoglobin (MetHb) contains hemoglobin that has been oxidized from its ferrous (Fe^2+^) to ferric (Fe^3+^) state [1–5]. Iron in the ferric state is unable to bind and transport oxygen to tissues [1, 2, 5]. Methemoglobinemia can be an acquired or congenital condition. The more common acquired form occurs from exposure to oxidative agents such as dapsone, benzocaine, nitrates, nitrites, and aniline derivatives [5–7]. While rarer, Hemoglobin M (HbM) variants cause a distinct congenital form of MetHb involving mutations in the α, β, and γ globin gene resulting in resistance to reduction by cytochrome B_5_ reductase (CYB_5_R). This case describes the presentation of a term infant with persistent cyanosis, found to have a rare familial γ-chain mutation. This unusual case emphasizes several diagnostic challenges inherent in neonatal hemoglobinopathies, highlights the typical time course for various methemoglobinopathies, and underscores the increasing importance of rapid whole genome sequence in diagnosing this rare cause of neonatal cyanosis.

## Case presentation

A 3-kg term male neonate developed cyanosis and hypoxia without signs of respiratory distress shortly after birth. His oxygen saturations were persistently 70–80% despite 1.0 FiO2 and increased respiratory support to CPAP+ 6 cm H_2_O. The initial physical exam was significant for acrocyanosis. Due to concern for congenital heart disease, he was transported to a Level IV neonatal intensive care unit (NICU) for further evaluation and management.

On admission to the NICU, he was quickly weaned from CPAP to low flow nasal cannula. An initial chest x-ray was normal. Pre- and post-ductal oxygen saturations were equal and remained below 85%. An initial echocardiogram demonstrated normal cardiac anatomy; capillary blood gas was reassuring with normal pH and an elevated pO2. Investigations to rule out hemolysis (complete blood count, reticulocyte count, peripheral smear, lactate dehydrogenase, bilirubin, haptoglobin, Heinz body preparation) and end-organ dysfunction (liver function tests, electrolytes, renal function tests) were within acceptable range.

Given the absence of a clear cardiac or pulmonary etiology of his persistent cyanosis, hematologic causes such as congenital or acquired methemoglobinemia were explored. Upon initial transfer, no family history was available, and attempts were made to obtain methemoglobin levels to confirm this diagnosis were unsuccessful due to unconjugated hyperbilirubinemia that interfered with laboratory measurement. Methylene blue and ascorbic acid treatments were considered, and hematology and medical genetics teams were consulted. However, upon arrival of the presumed biological father, a thorough history to evaluate for perinatal exposures was obtained and he reported an extensive paternal family history of neonatal cyanosis due to a rare mutation causing a HbM variant. There was no other family history of anemia or blood disorders. Given this new information, hematology recommended supportive care without any specific treatment, as well as further testing to confirm the diagnosis of this congenital methemoglobinopathy. The genetics team recommended whole genome sequencing which later revealed a likely pathogenic variation in HBG2(c.277C > T). He was trialed on room air several times and developed intermittent respiratory distress and fatigue with feeds, so was maintained on 0.25–0.5 L/min nasal cannula to help support feeding endurance during his hospitalization. His bilirubin levels were elevated > 5 mg/dl for the duration of his hospitalization (total serum bilirubin of 12.8 mg/dL on day of discharge, which was low intermediate risk). A methemoglobin level was never able to be directly obtained. The neonate was discharged home at 2 weeks of age on full oral feeds with 0.25 L/min nasal cannula as respiratory support, with close outpatient follow-up scheduled with hematology, genetics, and pulmonology. By 5 weeks of age, he had resolution of cyanosis and was successfully weaned off respiratory support.

## Discussion and conclusion

Methemoglobin describes the form of hemoglobin (Hb) in which the heme moiety contains iron that has been oxidized from its ferrous (Fe^2+^) to ferric (Fe^3+^) state [1–5]. This oxidized Hb is then unable to bind and release oxygen to the tissues. In normal erythrocyte metabolism, methemoglobin is continuously produced and then reduced enzymatically by nicotinamide adenine dinucleotide (NADH) cytochrome B_5_ reductase [7], and usually comprises < 1% of total Hb [2–4]. Methemoglobinemia occurs when the balance between oxidation and reduction reactions are disrupted, and methemoglobin accumulates to > 3% [4, 5].

The congenital form of MetHb can be caused by an autosomal recessive inheritance of a deficient CYB_5_R gene but can also be due to hemoglobin variants called HbM [6–10]. HbM are a group of defects due to single amino acid substitutions in the normal globin chain, inherited in an autosomal dominant fashion. In HbM, heme iron is stabilized in the ferric state and resists reduction by the normal enzymatic pathway [1, 2, 11]. These can occur in α, β, and γ globins and thus can affect both fetal and adult hemoglobin. There are several known variants of HbM, including Boston, Fort Ripley, Hyde Park, Iwate, Kankakee, Osaka, and Saskatoon, but the true incidence is not known [12, 13]. Patients with HbM variants usually have MetHb levels in the 30–40% range yet have few symptoms due to physiologic compensation over time [14].

Hb F-M-Fort Ripley (HBG2: c.227 C > T) is a form of HbM caused by a missense mutation in the gamma chain, causing a histidine residue at codon 92 to be replaced with tyrosine [10]. It is one of six known HbM γ-globin variants associated with neonatal cyanosis [4, 15, 16]. It was first described in a case report by Priest et al. in 1989 [7], and again by Molchanova et al. in 1992 [17]. It demonstrates autosomal dominant inheritance and incomplete penetrance [18].

The approach to the neonate with persistent cyanosis without evidence of a cardiac or pulmonary etiology should raise suspicion for methemoglobinemia. Acquired or congenital cases of MetHb that result in deficiency of CYB_5_R can be treated with methylene blue at a dose of 0.5–2 mg/kg administered intravenously over 5 minutes and repeated after 1 hour if there is insufficient reduction in MetHb [2, 5]. Ascorbic acid is an alternative treatment, utilized in instances where methylene blue is unavailable [19]. Treatment is typically reserved for symptomatic patients with methemoglobin levels > 10% [2, 5, 19]. While treatment with methylene blue is very effective in the setting of acquired or congenital methomoglobinemia, it does carry a risk of severe hemolysis in patients with Glucose-6-Phosphate Dehydrogenase (G6PD) deficiency and therefore warrants a detailed family history to assess for risk of G6PD deficiency, as well as testing for G6PD deficiency in patients anticipated to receive methylene blue [20]. It is not always feasible to obtain G6PD deficiency results prior to initiation of methylene blue treatment, which necessitates close post-treatment monitoring for signs of hemolysis, as this could be life-threatening.

While rarer, HbM is another congenital form of methemoglobinemia involving mutations in the α, β, and γ globin gene resulting in resistance to reduction by CYB_5_R. There is substantial variability in initial timing of cyanosis and persistence of cyanosis dictated by the specific globin gene mutated. In patients with mutations to the α globin gene, which is present in the predominant forms of hemoglobin at birth (fetal Hemoglobin F (HbF) [α2γ2) and throughout life (adult Hemoglobin A (HbA) [α2β2]), patients typically present with cyanosis in the neonatal period and can persist throughout life [1]. Patients with mutations in β globin genes typically do not present in the neonatal period, but rather present around 6 months of life as HbA (α2β2) takes over as the predominant form of hemoglobin and can persist throughout life [1–5]. Although mutations in the γ globin gene are more rare, these forms of HbM typically result in transient cyanosis that presents during the initial neonatal period and resolves within the first 6 months of life as fetal hemoglobin declines and is replaced by adult hemoglobin over time; it is not expected to recur [5, 7, 17].

Initial evaluation for methemoglobinemia in the neonatal period involves obtaining a methemoglobin level, which can help dictate if and when treatment should be initiated. However, obtaining a MetHb level in a neonate can be a challenge. Routine pulse oximetry is unable to distinguish between MetHb and normal oxyhemoglobin and deoxyhemoglobin [21, 22]. Co-oximetry specialized pulse oximeters that use multiple wavelengths of light can detect absorption of MetHb at 630 nm (6.3e-7 m) [16–18]. The most accurate method of detecting MetHb is the Evelyn-Malloy assay, but this specialized method of methemoglobin detection is rarely available in a timely fashion [17, 18]. Accurate quantification of MetHb is difficult in the presence of other substances that absorb light at around 630 nm. High concentrations of HbF, bilirubin, and lipids in neonatal patients can obscure results [20, 23, 24]. In our patient, the unconjugated bilirubin level was > 5 mg/dL (442 μmol/L) at 4 hours of life, which invalidated all available local methods for determining the concentration of MetHb.

In instances where methemoglobin levels cannot be obtained, studies evaluating CYB_5_R activity and G6PD deficiency are recommended. If there is concern for a Hemoglobin M, hemoglobin electrophoresis can be helpful to identify a hemoglobinopathy. Hemoglobin electrophoresis can be helpful in identifying variants to the α and β globin genes, however Hb F-M-Fort Ripley is unstable and variants to the γ globin gene are often not detectable via hemoglobin electrophoresis [7, 10, 17]. Therefore, in cases with high suspicion of Hemoglobin M due to a mutation in the γ globin gene further genetic testing to confirm diagnosis is warranted.

Follow up with hematology is dictated by the expected persistence of cyanosis based on the specific HbM variant. In general, patients with congenital methemoglobinemia can compensate well, often with an adaptive polycythemia and can tolerate methemoglobin levels up to 40% without significant symptoms. However, these patients are at high risk of acute decompensation when challenged with oxidizing agents [20].

Because Hb F-M-Fort Ripley is due to a problem with γ globin, symptoms are transient as HbF production converts to HbA production over the first few months of life [7, 10]. Prior case reports of infants with Hb F-M-Fort Ripley have described resolution of cyanosis between four and 8 weeks of life, or 42 weeks corrected gestational age in the case of a premature infant with concurrent bronchopulmonary dysplasia [7, 10, 15]. Of our patient’s affected family members, including the father and aunt among others [7, 17], the individuals were asymptomatic at birth, but developed cyanosis by a few days of age with no other major medical concerns. They had prolonged NICU stays for cyanosis and hypoxia, but none required treatment with methylene blue and none have had recurrent issues with hypoxia to date. Their cyanosis resolved by 3–8 weeks of age [7, 17]. Our patient was successfully weaned off supplemental oxygen by 5 weeks of age, with no recurrence of his cyanosis. For this patient, given the expected transient nature of his methemoglobinemia and cyanosis, he was seen for follow up in hematology clinic upon discharge from the NICU and at 2 months of life with resolution of his cyanosis noted at that time. No further hematology follow-up has been necessary, and no further attempts at obtaining methemoglobin levels have been made.

## Data Availability

Not applicable.

## Abbreviations

CYB_5_R: Cytochrome B_5_ reductase
G6PD: Glucose-6-Phosphate Dehydrogenase
Hb: Hemoglobin
HbA: Hemoglobin A
HbF: Hemoglobin F
HbM: Hemoglobin M
MetHb: Methemoglobinemia
NICU: Neonatal Intensive Care Unit
NADH: Nicotinamide adenine dinucleotide

## References

[1] Ludlow JT, Wilkinson RG, Nappe TM (2022). Methemoglobin. 2021 Sep 2. StatPearls [Internet].

[2] Da-Silva SS, Sajan IS, Underwood JP (2003). Congenital methemoglobinemia: a rare cause of cyanosis in the newborn--a case report. Pediatrics..

[3] Schwartz L, Franck P, Debruille C, Olivier JL, Vigneron C (2005). Congenital methemoglobinemia: a rare case of cyanosis in the newborn. Ann Biol Clin (Paris).

[4] Glader BE (1989). Hemoglobin FM-Fort Ripley: another lesson from the neonate. Pediatrics..

[5] Viršilas E, Timukienė L, Liubšys A (2019). Congenital methemoglobinemia: rare presentation of cyanosis in newborns. Clin Pract.

[6] DomBourian M, Ezhuthachan A, Kohn A, Berman B, Sykes E (2015). A 5-hour-old male neonate with cyanosis. Lab Med.

[7] Priest JR, Watterson J, Jones RT, Faassen AE, Hedlund BE (1989). Mutant fetal hemoglobin causing cyanosis in a newborn. Pediatrics..

[8] Lees MH, King DH (1987). Cyanosis in the newborn. Pediatr Rev.

[9] Percy MJ, Lappin TR (2008). Recessive congenital methaemoglobinaemia: cytochrome b(5) reductase deficiency. Br J Haematol.

[10] Ghotra S, Jangaard K, Pambrun C, Fernandez CV. Congenital methemoglobinaemia due to Hb F-M-Fort Ripley in a preterm newborn. BMJ Case Reports. 2016:bcr2016214381. 10.1136/bcr-2016-214381. 10.1136/bcr-2016-214381 PMC4800241 26969357

[11] Spears F, Banerjee A (2008). Hemoglobin M variant and congenital methemoglobinemia: methylene blue will not be effective in the presence of hemoglobin M. Can J Anaesth.

[12] Haymond S, Cariappa R, Eby CS, Scott MG (2005). Laboratory assessment of oxygenation in methemoglobinemia. Clin Chem.

[13] McGrath JS, Datir S, O’Brien F (2016). Why so blue? A case of neonatal cyanosis due to congenital methaemoglobinaemia (HbM Iwate). BMJ Case Rep.

[14] Curry S (1982). Methemoglobinemia. Ann Emerg Med.

[15] Hooven TA, Hooper EM, Wontakal SN, Francis RO, Sahni R, Lee MT (2016). Diagnosis of a rare fetal haemoglobinopathy in the age of next-generation sequencing. BMJ Case Rep..

[16] Hayashi A, Fujita T, Fujimura M, Titani K (1980). A new abnormal fetal hemoglobin, Hb FM-Osaka (alpha 2 gamma 2 63His replaced by Tyr). Hemoglobin..

[17] Molchanova TP, Wilson JB, Gu LH (1992). A second observation of the fetal methemoglobin variant Hb F-M-Fort Ripley or alpha 2G gamma 2(92)(F8)His----Tyr. Hemoglobin..

[18] Hain RD, Chitayat D, Cooper R (1994). Hb FM-Fort Ripley: confirmation of autosomal dominant inheritance and diagnosis by PCR and direct nucleotide sequencing. Hum Mutat.

[19] Rino PB, Scolnik D, Fustiñana A, Mitelpunkt A, Glatstein M (2014). Ascorbic acid for the treatment of methemoglobinemia: the experience of a large tertiary care pediatric hospital. Am J Ther.

[20] Fanaroff MCW (2019). Fanaroff and Martin’s neonatal-perinatal medicine: diseases of the fetus and infant.

[21] Ward J, Motwani J, Baker N, Nash M, Ewer AK, Toldi G (2019). Congenital Methemoglobinemia identified by pulse oximetry screening. Pediatrics..

[22] Chan ED, Chan MM, Chan MM (2013). Pulse oximetry: understanding its basic principles facilitates appreciation of its limitations. Respir Med.

[23] Lampert R, Brandt L (1993). The effect of hyperbilirubin in the measurement of oxygenated Hb (O2Hb), carboxyhemoglobin (COHb) and methemoglobin MetHb using multiwavelength oximetry in mixed venous blood. Anaesthesist..

[24] Speakman ED, Boyd JC, Bruns DE (1995). Measurement of methemoglobin in neonatal samples containing fetal Hb. Clin Chem.

